# Constraints on Negative Prefixation in Polish Sign Language

**DOI:** 10.1371/journal.pone.0143574

**Published:** 2015-11-30

**Authors:** Piotr Tomaszewski

**Affiliations:** Faculty of Psychology, University of Warsaw, Warsaw, Poland; Kyoto University, JAPAN

## Abstract

The aim of this article is to describe a negative prefix, NEG-, in Polish Sign Language (PJM) which appears to be indigenous to the language. This is of interest given the relative rarity of prefixes in sign languages. Prefixed PJM signs were analyzed on the basis of both a corpus of texts signed by 15 deaf PJM users who are either native or near-native signers, and material including a specified range of prefixed signs as demonstrated by native signers in dictionary form (i.e. signs produced in isolation, not as part of phrases or sentences). In order to define the morphological rules behind prefixation on both the phonological and morphological levels, native PJM users were consulted for their expertise. The research results can enrich models for describing processes of grammaticalization in the context of the visual-gestural modality that forms the basis for sign language structure.

## Introduction

This paper seeks to contribute to the body of research on Polish Sign Language (PJM) by focusing on morphology and phonology. In particular, this work focuses on a PJM *negative prefix*. The analysis of the PJM negative prefix may shed new light on the morphophonological constraints governing the manner of articulation of this type of morpheme. In addition, it could enrich the theory of grammaticalization in the sequential morphology of sign languages.

Research conducted on the sequential morphology of sign languages to date shows that processes of affixation also take place in other sign languages [1–3]. In previous research more suffixes than prefixes have been identified in sign languages. Nevertheless, Aronoff, Meir and Sandler [4] have shown that Israeli Sign Language (ISL) has sensory prefixes that will be discussed later in this article. Zeshan [5, 6] noted that in contrast to spoken languages, sign languages make use of different morphological means of negation with a negative morpheme attached to the predicate: “They are exclusively suffixing, with no attested case of a negative prefix in our data” (see Results in [5]). As it turns out however, there is one negative prefix in PJM that appears to be indigenous to the language, and has been grammaticalized from an independent sign.

The article will focus on (1) the theoretical background of sign language morphology in terms of simultaneity and sequentiality; (2) the structure of the independent fingerspelled sign #nie and the signed prefix neg- which is derived from it; (3) the constraints on the use of the negative prefix and (4) the comparison of sequential compounding and negative prefixation in terms of the constraints operating on them.

## Sign Language Morphology

Sign language structure includes sequences of movements and configurations of the hands or arms, the face, head and torso. In contrast to sign languages, spoken languages are made up of sounds arising out of a sequence of movements and configurations of the supralaryngeal vocal tract as well as by air flowing through the vocal folds. Sign languages are therefore visual-gestural, while spoken languages are auditory-vocal in nature. Despite this difference, which is due to the differing language modalities, similarities between sign and spoken languages are also found. This is because both have similar phonological, morphological and syntactic subsystems [1,7–10]. Therefore, natural sign languages are formally structured at different levels and follow the same universal constraints and organizational principles of all natural languages [1,11]. Given the subject matter of this paper, however, the focus here will be particularly on the morphological aspects of sign languages.

There are two types of morphology in sign languages: simultaneous and sequential. The first is preferred in the sign modality and refers to a simultaneous combination of morphemes, meaning that the expressions of morphemes overlap. That is to say, the meaningful units are added not by adding segments but rather by changing their internal structure. For a similar example in a spoken language, the singular form of the English word *m* ***a*** *n* forms the plural by changing the vowel (/æ/ to /e/) rather than by attaching the morpheme *-s* to the end of the root as happens with *pen*+*s*. Simultaneous processes in sign languages are implemented by changing the movement features of the sign; they may be inflectional or derivational in nature; their operations are numerous; they are related to spatial and temporal cognition, and most of them are non-arbitrary to various degrees; most important of all, they are not grammaticalized from free signs [2,4,12].

In contrast, the sequential type, which consists of sequential combinations of morphemes is less preferred in the sign modality. Sequential morphology in sign languages is quite similar to its spoken language counterpart in that the elements in a sequence (words and affixes) are signed one after another in linear order (like the complex word *fullness* in English). Each of the elements, as a morpheme, has a complete set of phonological segments, which, in sign languages, relate to the complete specifications for the formational units of handshape, orientation, location and movement. Sequential operations in different sign languages are only derivational and relatively sparse. They also tend to be more arbitrary; most importantly, affixes have been grammaticalized from free-standing signs [2,4,12].

This paper will focus on sequential affixation in sign languages. While sequential affixation takes place in sign languages, it is rare and may be due to the effect of the visual-gestural modality preferring simultaneous constructions rather than sequential ones. Still, Aronoff et al. [12] suggest that because the grammaticalicization of affixes takes time and sign languages are relatively young, their modality might be responsible for the limited nature of their affixal morphology. Grammaticalization is a complex set of diachronic processes by which lexical morphemes in a language change over time and become grammatical morphemes or to put it another way, morphemes that are less grammatical in nature develop into ones that are more grammatical [13]. This process involves the following interrelated mechanisms: (1) semantic bleaching–loss in meaning content; (2) extension–use in new contexts; (3) decategorialization–loss of morphosyntantic properties characteristic of lexical or other less grammaticalized forms; (4) phonological erosion–loss in phonetic substance [14]. One of the ways of identifying whether particular units are affixes or compound members is analyzing the degree of productivity in these units. Morphological productivity is the extent to which a morphological pattern can be applied to new forms [15]. A well-known example of a productive derivational suffix in English is–*ness*, which is much more productive than the compound member *green* [2]. Nonetheless, different degrees of productivity are also observed in the affixes themselves. Some of them may be more productive than others. In any case, the degree of productivity among affixes and compound members is determined by the fact that affixes are bound morphemes that must combine with other morphemes to form a structure constituting a word, whereas compound members can function independently as free words and can be combined into single words with fewer morphemes.

The morphological system of a sign language comprises—as is the case with spoken language—both a set of productive means that make up an active morphological subsystem and a set of non-productive means that are a passive morphological subsystem. One of the morphological elements that can expand the PJM lexis is, first of all, the negative prefix NEG- discussed in this paper. This prefix can be creatively merged with many morphemes functioning as verbs or adjectives. On the other hand, its suffixal counterpart, the suffix -NEG has a similar function but is not productive. It is worth noting that this unproductive suffix has a very similar counterpart in American Sign Language (ASL). The PJM suffixed sign ZNAĆ+NEG ‘not know’, for example, has the same form as the ASL sign DON’T-KNOW. This affixation process has been termed negative incorporation by Woodward [16]. It was claimed this negative suffix is thought to derive from the French Sign Language sign NOT [17]. This negative bound morpheme may be affixed to several signs (e.g. KNOW, LIKE, WANT, HAVE and GOOD) to form a negative instead of using separate negative signs (NOT, DON’T).

To date, a number of studies have been conducted on sequential affixation, in particular on concatenative derivation in different sign languages. The findings reveal that sign languages also exhibit prefixes and suffixes. In ASL, for example, suffixes for comparative and superlative (e.g. GOOD + comparative, GOOD + superlative) and an agentive suffix (e.g. LEARN + agentive, TYPE + agentive) have been found [1]. ASL also has a suffix–ZERO ‘not (verb) at all’ (e.g. TOUCH+ZERO ‘not use’, UNDERSTAND+ZERO ‘not understand at all’) which appears to have been grammaticalized from an independent sign with a similar meaning [4,18]. Similarly, ISL has a class of affixes, a set of ‘sense’ prefixes and a negative suffix NOT-EXIST [4,12,18,19]. These prefixes can be attached to nouns, verbs, and adjectives. They seem to be have developed from signs that refer to sensory perception or cognition. They can be used to make forms that usually have meanings similar to 'to do something by seeing/smelling (intuiting)' " (e.g. EYE+SHARP 'discern through sight', EYE+CATCH) [12]. The ISL negative suffix means ‘without’, which is similar in meaning to the English suffix–*less* (e.g. INTEREST+NOT-EXIST ‘without interest’, SUCCESS+NOT-EXIST ‘without success, unsuccessful’). This morpheme was apparently grammaticalized from a negative sign meaning roughly ‘none’. There is evidence that negative suffixes appear in many different sign languages besides ASL and ISL. For example, British Sign Language (BSL) has a negation sign which can be attached to a verb as a sort of suffix, e.g. SEE + neg, or HAVE + neg [7,20]. As Sutton-Spence and Woll (see Results in [7])] note, “It is often used for denial of possession, presence, or experience”. Affixation has also been found in Chinese Sign Language [21], Finnish Sign Language [5], Greek Sign Language [22], Australian Sign language [8] and Jordanian Sign Language [23]. In these languages, the negative particle can used as a suffix in order to construct a sign with a negative meaning. Two potential suffixes have recently been found in Al-Sayyid Bedouin Sign Language. These are locative pointing signs [24,25] and size and shape signs [25,26]. However, research in this area is still ongoing because it is difficult to determine whether these morphemes are affixed signs or compounds [2].

The above-described negative suffixes in these sign languages and ‘sense’ ISL prefixes are argued to be affixes rather than independent signs for several reasons: (1) These affixes are productively attached to lexical items; (2) Signs cannot be inserted between them and the base they attach to; (3) They must precede their base; (4) Some affixed signs show idiosyncrasies in form and meaning; (5) There are some arbitrary gaps in the lexical items that can take the affixes, and (6) some base signs (e.g. verbs) and affix forms (in particular the suffix–ZERO) are fused phonologically [1,4,12,18]. Hence, all sign languages appear to allow negative suffixation for a semantically similar set of verbs related to personal experience.

As it turns out, PJM also has a set of affixes, including the negative prefix NEG- which is productively attached to many signs. Although, there is a group of signs which cannot combine with the morpheme NEG-. The remainder of this paper focuses on the following research questions: What are the *constraints* on affixation of the morpheme NEG-? What is the nature and source of the constraints on affixation of the morpheme NEG-? Are there any similarities or differences between constraints on affixation and constraints on compounding? Before these questions are examined, a preliminary description of the source of the morpheme NEG- in PJM will be presented.

## The Case of the PJM Negation Prefix NEG-

Before describing the process by which the negative prefix NEG- in PJM emerged, the fingerspelled loan sign #NIE and the context, in which it is used should be presented. This type of loan sign reflects a process of lexicalization that appears with the borrowing of fingerspelled lexical units from written Polish. This particular case is a good example of the erosion of structural transparency that occurs with some loan signs.

### The fingerspelled loan sign #NIE ‘no/not’

In fingerspelling, the letters of written Polish words are represented by the different hanshapes of the Polish manual alphabet which is used by PJM signers to "spell" proper names (especially personal and place names), technical vocabulary, other words not associated with particular signs as well as abbreviations and acronyms.

The lexical stock of PJM includes non-native signs such as, inter alia, fingerspelled signs borrowed from written Polish through the Polish manual alphabet. The presence of Polish loans in PJM is the result of the continuous contact between the two languages. Polish loan signs are abbreviated fingerspelled units. They are not calques but are rather loanwords taken into PJM from Polish in a changed form, relating to the nature of sign phonology and morphology. Fingerspelled loan signs in PJM have their own segmental structure and internal parameters such as handshape, location, movement and orientation. From the morphological point of view fingerspelled loans in PJM are independent morphemes.

**Fig 1:**
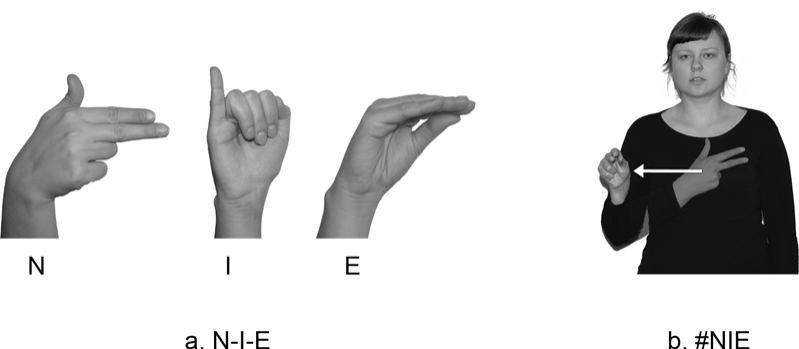
Fingerspelled versions of NIE ‘no’: N-I-E and #NIE. The individual in this figure has given written informed consent (as outlined in PLOS consent form) to publish these case details.

In his description of lexicalization, Battison [27,28] distinguishes several ways, in which fingerspelled loan signs borrowed from English into ASL may change their form (see S1 Table). His conclusions, in reference to ASL, may be transferred to PJM, in which there is a process of lexicalization of loanwords from the surrounding spoken and written language. An example of this is the fingerspelled loan sign #NIE which is related to the Polish particle of negation *nie* 'no/not'. Fig 1 shows the fingerspelled versions of NIE ‘no/not’: N-I-E and #NIE:

Fig 1A indicates how it is possible to fingerspell N-I-E letter by letter, articulating three fingerspelling letters -N-, -I- and -E-. Fig 1B on the other hand shows the way, in which the lexicalized loan #NIE is articulated. During the process of lexicalization, the fingerspelling letter -I- is omitted so that #NIE uses only two fingerspelling letters -N- and -E-. It is articulated in such a way that the initial -N- and the final -E- are fingerspelled and the medial letter -I- disappears. The next step in the process of lexicalization of the loan #NIE is a change in the handshape. The sign has two handshapes, (1) the shape of the letter -N- which consists of an extended thumb and two fingers (the index and middle ones) that are extended and held apart, and (2) the letter -E- which is indicated by the ring and little fingers curled while the other fingers are extended, touching each other. In isolation, both letters are articulated differently as the two fingers are not spread for -N-, while for -E-, all the extended fingers are touching each other. So, the fingerspelled loan sign #NIE is made by a modified version of the letter -N- formed in front of the middle of the chest. The hand then moves to the ipsilateral side of the signer while changing into the modified -E- and the fingertips (of the thumb, index and middle fingers) point away from the signer [29,30].

Within the Hand Tier model of Sandler [31,32], the sign #NIE has one syllable with the structure of Location-Movement-Location (LML) as it is made up of three segments, two locations and one path movement. While during the movement from the first to the second location parameter of handshape undergo changes as does the parameter of orientation (palm → area of the thumb) (see Fig 1B).

**Fig 2:**
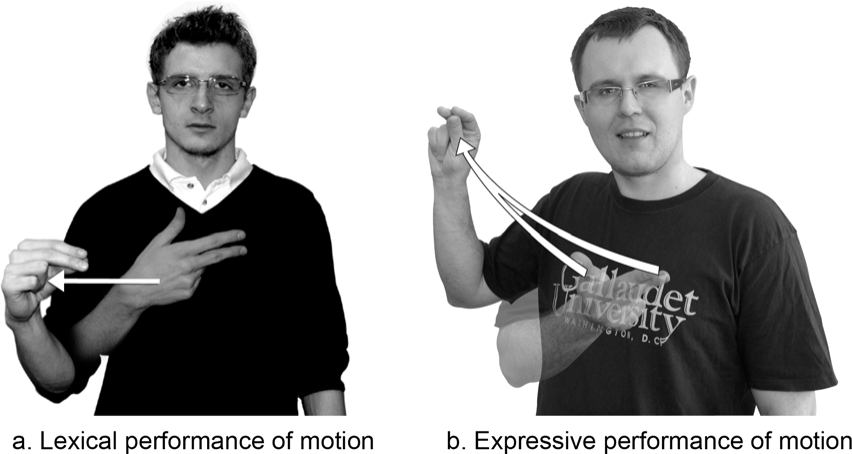
Two ways of articulating the fingerspelled loan sign #NIE: lexically or expressively. The two individuals in this figure have given written informed consent (as outlined in PLOS consent form) to publish these case details.

While the articulation of #NIE has been described above, it is necessary to show in what contexts it may be used. The negator #NIE can be used as a whole utterance, for example as an answer to a question. At the pragmatic level, the sign #NIE is often used alone to give a negative answer. Also, #NIE may take on a prosodic feature. Some PJM signs may undergo an energetic articulatory lengthening which has an expressive value. There is a similar process in Polish where a vowel or consonant can be lengthened for expressive purposes. As Fig 2 shows, the negator #NIE may, under certain emotional conditions, be elongated, corresponding prosodically to the Polish negator *Nieee*! 'Nooo!' (where the repeated letter indicates lengthening).

At the level of syntax, the fingerspelled loan sign #NIE can be used in PJM negative sentences which occur with a rhetorical question and which may play the role of single sentences ([33]; see S1 Text). Additionally, the sign #NIE can function sententially as a negative imperative sign with stress on the movement and a slight forward tilt of the body and head.

This section illustrates the formation of a negative prefix on the basis of the sign #NIE, combined only with certain lexical units. This phenomenon is a process in which a construction that contains a particular lexical item becomes grammaticalized. Moreover, it has a sequential structure that differs significantly from the simultaneous type.

### The emergence of morpheme NEG- in the process of prefixation

**Fig 3:**
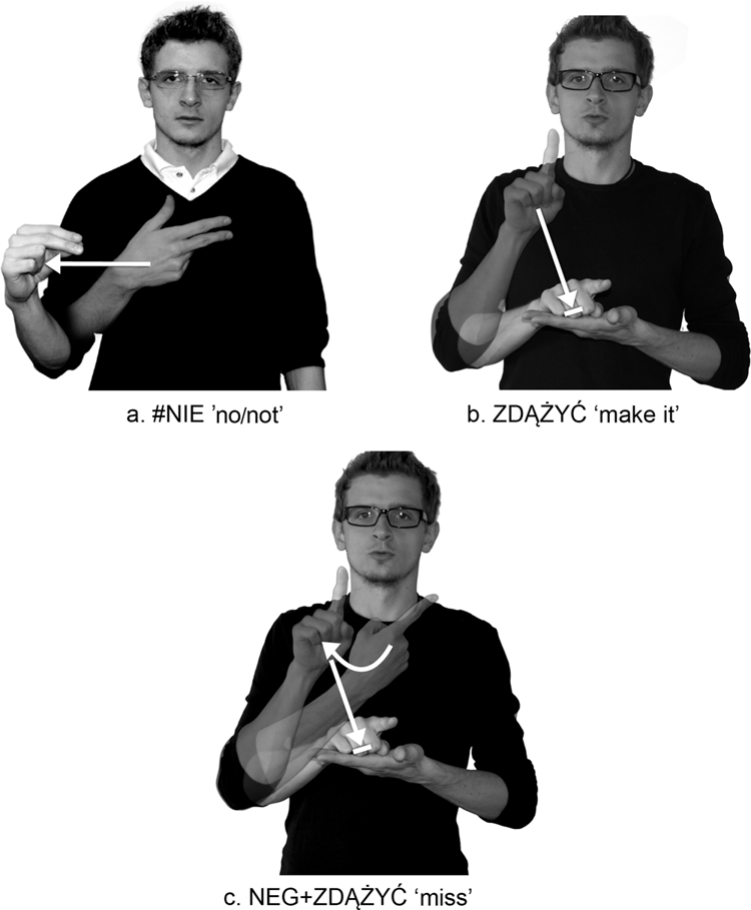
PJM sign ZDĄŻYĆ ‘make it’ with the grammaticalized negative prefix NEG-. (A) the lexicalized loan #NIE (B) the lexical base ZDĄŻYĆ ‘make it’ (C) prefixed sign NEG+ZDĄŻYĆ ‘miss’. The individual in this figure has given written informed consent (as outlined in PLOS consent form) to publish these case details.

**Fig 4:**
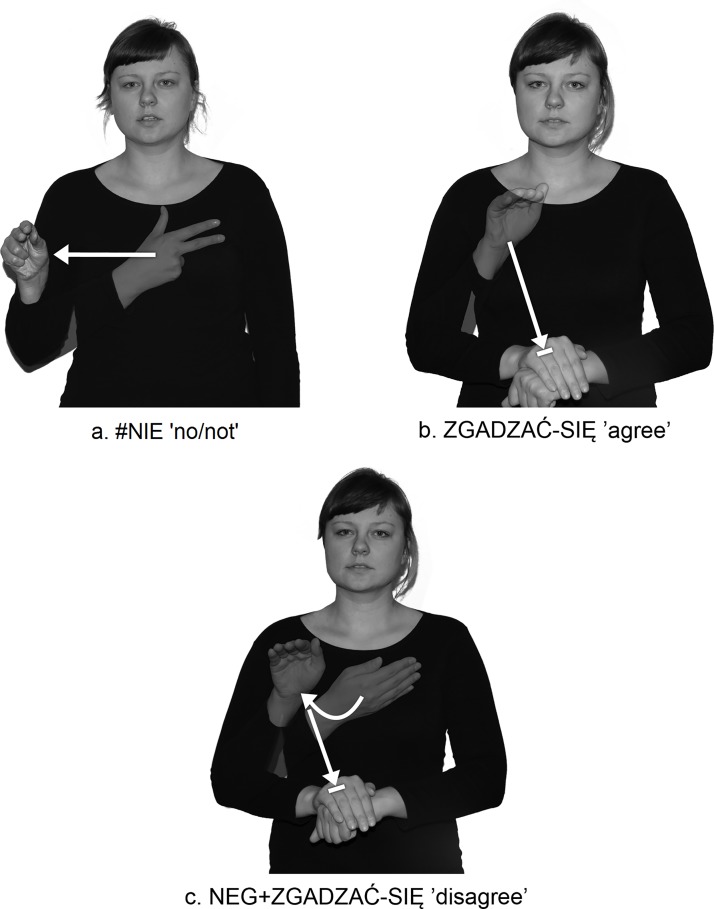
PJM sign ZGADZAĆ-SIĘ ‘agree’ with the grammaticalized negative prefix NEG-. (A) the lexicalized loan #NIE (B) the lexical base ZGADZAĆ-SIĘ ‘agree’ (C) prefixed sign NEG+ZGADZAĆ-SIĘ ‘disagree’. The individual in this figure has given written informed consent (as outlined in PLOS consent form) to publish these case details.

PJM has many prefixed signs, in which one morpheme is the negative prefix, while the other is a lexical item. Examples of such "fossilized" signs in PJM include NEG+ZDĄŻYĆ ‘miss’ (Fig 3) and NEG+ZGADZAĆ-SIĘ ‘disagree’ (Fig 4, S1 Fig), where the first morpheme includes the negative prefix, glossed as NEG-, and the other is lexical ZDĄŻYĆ ‘make it’ and ZGADZAĆ-SIĘ ‘agree’.

As mentioned above, the NEG- prefix is derived from the fingerspelled loan sign #NIE ‘no/not’, which is an independent morpheme. This morpheme, when combined with the verbs ZDĄŻYĆ ‘make it’ or ZGADZAĆ-SIĘ ‘agree’, becomes the derivational morpheme NEG-. This morpheme cannot appear separately, and must be prefixed to other lexical items.

According to the criteria proposed by Zwicky and Pullum [34], the prefix NEG- appears to have the properties of an affix. Firstly, the prefix can be used with two sign categories, i.e. verbs and adjectives, but cannot be used with nouns (while there does not seem to be a formal distinction between predicate adjectives and verbs in most sign languages the term adjective is used here for ease of understanding). So, there is evidence that the negative prefix NEG- represents a single grammatical category. When used with adjectives, the negation prefix is similar to the English prefix *un–*. Some examples are presented in Table 1 (the PJM prefixed signs are used by Deaf signers in S1–S8 Videos):

**Table 1:** Examples of prefixed PJM signs as negated verbs or adjectives.

negated verbs	negated adjectives
NEG+ZDĄŻYĆ ‘miss’	NEG+WINNY ‘not guilty/innocent’
NEG+ZGADZAĆ-SIĘ ‘disagree’	NEG+WYGODNY ‘uncomfortable’
NEG+DAĆ ‘not give’	NEG+POTRZEBNY ‘unnecessary’
NEG+ŻYĆ ‘not alive’	NEG+SYMPATYCZNY ‘unlikeable’

**Fig 5:**
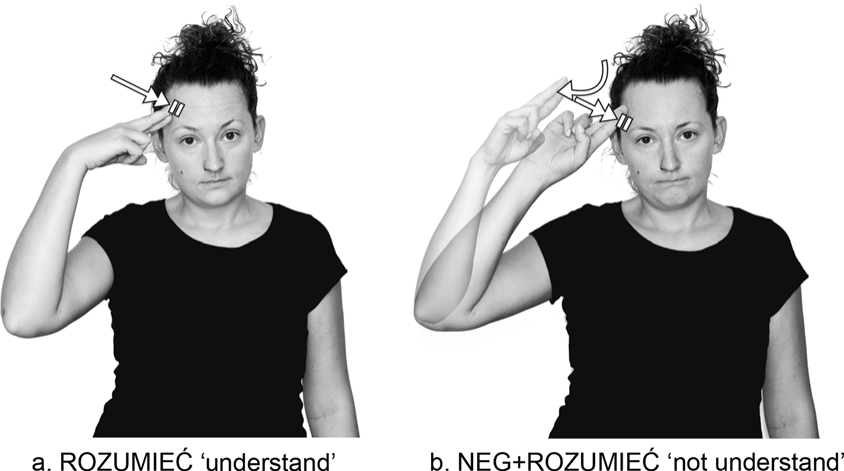
Handshape and location assimilation in the prefixed sign NEG+ROZUMIEĆ. (A) the lexical base ROZUMIEĆ ‘understand’ (B) prefixed sign NEG+ROZUMIEĆ ‘not understand’. The individual in this figure has given written informed consent (as outlined in PLOS consent form) to publish these case details.

Secondly, the form of the NEG- prefix depends on the shape of the stem. In the process of prefixation, the handshape of the negation prefix NEG- is assimilated to the handshape of the lexical item to which the prefix is attached (Figs 3 and 4, S1 Fig). Also the location of NEG- is partly assimilated with the location of the stem. The full assimilation in terms of handshape and location is found, for example, in the prefixed sign NEG+ROZUMIEĆ ‘not understand’ (Fig 5B, S9 Video).

In combining the two signs, #NIE and ROZUMIEĆ ‘understand’, the two sequential handshapes that make up #NIE are completely assimilated to the “R” handshape of ROZUMIEĆ ‘understand’ (Fig 5). At the same time, the location of the first sign loses the feature [chest] and assumes the feature [head] used by the second sign. In addition, in the prefixed sign NEG+ROZUMIEĆ ‘not understand’, the hand orientation is assimilated, too. In the separate sign ROZUMIEĆ ‘understand’, the orientation has the feature [fingertips]. But the prefix NEG- takes on the orientation feature [thumb area] at the end location. During the process of prefixation, the orientation of the morpheme ROZUMIEĆ ‘understand’ changes under the influence of the orientation of the final NEG- morpheme location. That is, the second morpheme loses the feature [fingertips] and takes on a different feature [thumb area] instead.

Moreover, when combined with a lexical item, the negative prefix tends to fuse phonologically as follows: The path movement of the prefix is shortened and has different shape features (arc and convex) than those of the morpheme #NIE with straight-line movement. So, the prefixed sign NEG+ROZUMIEĆ ‘not understand’ includes a sequence of two different motions, arc movement + restrained double movement (S2 Fig). There are PJM signs with the movement feature [restrained], where the movement “has the phonetic effect of doubling the pronunciation of the sign” (see Results in [35]). As Sandler [35] noted (for ASL), this feature refers to shortened and doubled movement, and occurs in signs that are lexically reduplicated (see the PJM sign ROZUMIEĆ ‘understand’ in Fig 5A).

It should be noted that this sequence of movements indicates a process of affixation, not of reduplication, that is, a process of forming new signs by doubling the whole free morpheme. This is supported by the following reasons: (1) While reduplication is used in sign languages to refer to the repetition of the movement segment in a sign, this does not apply to the way, in which prefixed PJM signs attach the NEG- affix to some lexical signs, and in addition, this indicates that the movement of the NEG- affix is different from the movement in such signs; (2) Reduplication takes place after the free morpheme, which does not apply to the morpheme NEG- when it appears before the lexical morpheme.

Thirdly, the semantics of the resulting prefixed signs which take the NEG- prefix are not always predictable. Some signs have idiosyncratic meanings. For example, the phrase NEG+WYGODNY [NEG+COMFORTABLE] (S6 Video) does not always mean literally ‘uncomfortable’ but can also have the meaning, ‘to feel awkward’; equally, the meaning of NEG+DAĆ [NEG+GIVE] (S3 Video) is closer to ‘not fix somebody up with something’ rather than to ‘not give’.

The four arguments mentioned so far show that NEG- is not a free form but rather a negative prefix occurring as part of a complex sign. On account of that fact the PJM negative prefix occurs in such a productive manner with so many base signs, a list of prefixed signs taking this morpheme was composed to determine whether there are any restrictions on attaching the prefix NEG- to base signs. As it turns out, there are lexical morphemes, to which the morpheme NEG- cannot be attached. Hence the next section presents the attested constraints on the negative prefix in PJM.

Apart from the four arguments described above, it could be added that the NEG- prefix has two more features characteristic of affixes: (1) Prefixed forms cannot be interrupted by other signs, and (2) There appear to be arbitrary gaps in the list of verbs and certain adjectives that can affix the PJM prefix NEG- to form negatives.

It has been pointed out that the occurrence of prefixed signs in PJM is associated with the linguistic interference between PJM and the Polish–influenced signing known as “Signed Polish” or, as Wojda [36] referred to it, “Speech and Signed System” (SJM: *system jezykowo-migowy*). As previously mentioned, Polish has a particle of negation *nie* which occurs before personal verb forms (e.g. *nie zgadzam się* ‘I do not agree’, *nie rozumiem* ‘I do not understand’, *nie dam* ‘I will not give’), and which is also used with adjectives (*nieuczciwy* ‘dishonest‘, *niesympatyczny* ‘unlikeable’, *niewinny* ‘innocent’). Thus, the category of negation includes phrases that are directly negated–verbal constructions whose head is preceded by the particle *nie*. In SJM, verbs are negated in a similar fashion, e.g. N-(I)-E ROZUMIEĆ ‘not understand’, N-(I)-E ZGADZAĆ SIĘ ‘disagree’. Here, the articulation of the particle N-(I)-E differs from that of the fingerspelled loan #NIE (in SJM, either all the letters of the Polish word *nie* are fingerspelled or only the fingerspelling letter -I- is omitted). While this structure was originally carried over to PJM as a loan translation (calque), the phrases that included it underwent phonological and morphological changes, becoming prefixed words with the negator NEG- which always precedes the verb or adjective. It can be said that the PJM prefix NEG- has evolved under the influence of the ambient Polish (as well as signed Polish), and so this negative affix, and especially prefixed signs can be regarded as Polish loan translations and, consequently, a result of language contact.

## Materials and Methods

The material for this article came from a corpus of original sign texts produced by 15 Deaf native and near-native signers aged between 25 and 40 years of age (11 Deaf native and 4 near-native signers; 8 women and 7 men). "Deaf" with a capital letter refers to the social group that uses sign language. The members of the group share similar experiences, beliefs and cultural identity. Deaf native signers are those who have Deaf parents from whom they acquired sign language from birth, whereas Deaf near-native signers are (adult) Deaf children of hearing parents who encountered and acquired sign language from their peers in early childhood (from three to five years of age). The collected material added up to about 30 hours of recordings of casual conversations with Deaf people. These conversations were conducted individually in PJM with each Deaf person whose side of the conversation was recorded with a high resolution digital camera. The conversations were led by Deaf native signers trained in conducting interviews. Apart from some general guidance in terms of topics, the conversations recorded were natural without overt elicitation. The conversations touched on topics such as the cultural development of Deaf people around the world, the role of PJM in the Deaf community, cultural and social integration between Deaf and hearing people, various forms of activism for the Deaf community in Poland, Deaf sports, Deaf education and similar topics.

Ethical approval was granted by the Ethics Committee of the Faculty of Psychology,

University of Warsaw, prior to recruiting participants. Before the interviews, individual meetings with the participants were held. They were informed about the voluntary nature of their participation in the study and about their right not to participate. More importantly, after some recordings of their signing had been already collected for the present article, the participants were informed that excerpts from the recordings would be made publicly available. Consent to be interviewed (and recorded with a digital camera) was obtained from each participant. All individuals in this manuscript have given written informed consent (as outlined in PLOS consent form) to publish these case details.

This collected material was crucial for the analysis of the linguistic function of prefixed signs. The collected material from the interviews with Deaf people includes about 50 examples of different lexical units with negative prefixes excerpted from the PJM corpus (The PJM corpus belongs to the Polish Sign Language and Deaf Communication Research Laboratory of the Faculty of Psychology at Warsaw University). These examples are listed with data on the frequency of the various types of negation in S2 Table and S3 Fig. Consultations were also carried out with native PJM signers who had robust judgments about PJM constructions and the ungrammatical forms (*NEG+signs). Their expertise was helpful in identifying those verbs and adjectives which can take the prefix NEG- as well as constraints on the use of negative morphemes in PJM. Twenty nine examples of prefixed signs (S1–S29 Videos), some of which are described in the article, are included in the "Supporting Information" section.

## Analysis

Analysis of the negative prefix in PJM demonstrates that there are two types of constraints on the NEG- morpheme. These are (1) constraints connected with the occurrence of certain types of irregular negatives; and (2) morphophonological constraints on the application of NEG- prefixation.

### Certain types of irregular negatives vs negative prefix

**Fig 6:**
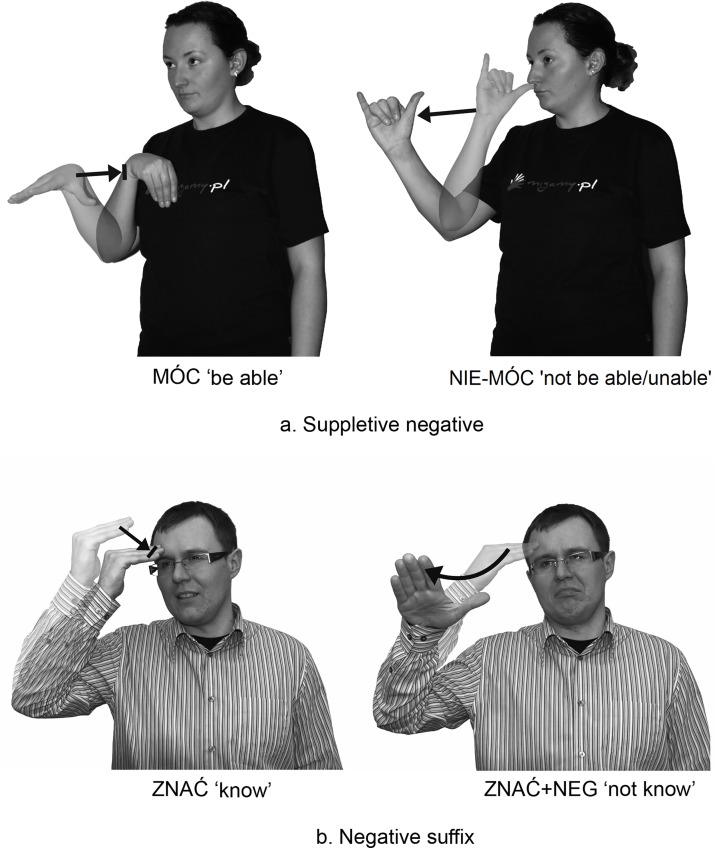
Examples of a suppletive negative and a negative suffix. The two individuals in this figure have given written informed consent (as outlined in PLOS consent form) to publish these case details.

There is a restriction concerning the occurrence of two types of irregular negatives in PJM, negative suppletion and the negative suffix. Indeed, these types of irregular negatives are limited to a few signs in PJM but, interestingly, if a PJM sign has a suppletive negative form or a negative suffix, then the prefix NEG- cannot be attached to their positive counterparts. It was noted that in many different sign languages there is a small set of verbs that have special negative marking attached to them (which is also true for PJM) [1,5,7,8,9,16,20]. Although this kind of marker seems to be a negative suffix, it is not productive according to these authors and is more integral to the sign base than is the case with a suffix. On the other hand, the PJM “underspecified suffix” -NEG complies with the criteria proposed by Zwicky and Pullum [34]. It is worth noting that the set of verbs that take this suffix differs across languages. However one verb does take this suffix in PJM, ASL and ISL. This is the sign ZNAĆ+NEG ‘not know’ in PJM (Fig 6B) and the sign NOT-KNOW in ASL (see [37]) and ISL (see [9]). The sign ZNAĆ or KNOW can be negated with this underspecified marker by adding an orientation rotation and a downward movement to the sign. Such negation, as previously mentioned, is termed negative incorporation [16]. However, the special negative marking hypothesis requires further research to determine the function of the negative marker.

Examples of types of irregular negatives are provided in Table 2.

**Table 2:** Comparison of selected types of irregular negatives.

Positive counterparts	Negative counterparts		
	suppletion	suffix	prefix
LUBIĆ ‘like’	NIE-LUBIĆ ‘not like’	——	——
MÓC ‘be able’	NIE-MÓC ‘not be able/unable’	——	——
UMIEĆ ‘be good at something’	NIE-UMIEĆ ‘not be good at something’	——	——
ZNAĆ ‘know’	——	ZNAĆ+NEG ‘not know’	——
CHCIEĆ ‘want’	——	CHCIEĆ+NEG ‘not want’	——
WIDZIEĆ ‘see’	——	WIDZIEĆ+NEG ‘not see’	——

In accordance with the rule on negative suppletion, the prefix NEG- cannot be combined with the phrase MÓC ‘be able’ to which negative suppletion regularly applies. Negative suppletion involves negative forms that are completely different from the corresponding non-negative forms. For example, Fig 6A shows an example of the negative suppletive form NIE-MÓC ‘not be able/unable’ and its non-negative counterpart MÓC ‘be able’ from PJM. Their parameters (handshape, location, movement, and orientation) differ. The two forms do not appear to be related to each other by any formational process.

The same occurs with the negative suffix. As Fig 6B shows, the negative suffix, which attaches to the base sign ZNAĆ ‘know’, consists of an outward twist of the wrist ending in an open “B” handshape. During the process of suffixation, the path movement of ZNAĆ ‘know’ is lost in the negative form that has the structure of a single sign rather than a two-sign combination. Interestingly, this negative suffix does not have any historical relation to the negative prefix NEG- discussed here. The negator NEG- originates from the fingerspelled version of #NIE which does not occur in the first dictionary of old PJM–the earliest recorded version of PJM as presented in the 1879 publication of the *Dictionary of mime for deafmutes and those having contact with them* assembled by two priests, Józef Hollak and Teofil Jagodziński [38]. The publication contains a description of the negative imperative sign NIE ‘no’ that belongs to the native lexicon of old PJM. While producing this sign, the head shakes and the hand is held farther from the body. It seems possible that the negative sign NIE ‘no’ was attached to the sign ZNAĆ ‘know’ which may be related to the current suffixed verb ZNAĆ+NEG ‘not know’.

### Phonological and morphological constraints on the negative marker NEG-

In order to uncover and formulate morphophonological constraints on the use of the marker NEG-, it is necessary to analyze two groups of signs: (1) signs to which NEG- can be added in a productive manner and (2) signs to which NEG- cannot be attached. Examples of signs in each group are given in Table 3 (the PJM prefixed signs listed in Table 3 were used by Deaf signers in S10–S28 Videos), and an analysis was carried out on all the signs. It should be emphasized that the first group of signs (NEG+signs) consists of both fossilized and feasible negative counterparts. Fossilized negative counterparts refer to those prefixed signs that are “indivisible”, i.e. they cannot be divided and used as separate signs (in this case, into the negator #NIE and a lexical morpheme). Among other elements, fossilized negative counterparts also include the prefixed sign NEG+ROZUMIEĆ ‘not understand’ (Fig 5B), because it is not possible to sign the two signs separately, #NIE and ROZUMIEĆ ‘understand’ as an alternative. Separate use of the sign #NIE before this verb is ungrammatical due to the strong assimilation between NEG- and the verb. Feasible negative counterparts are prefixed signs that can be optionally articulated as two separate signs: #NIE and the lexical unit. An example of a feasible negative counterpart would be the possible sign NEG+KOMENTOWAĆ ‘not comment’, apart from which it is also possible to use, as the alternative, the verb with a separate negative: #NIE KOMENTOWAĆ. The fossilized forms were noted directly from a corpus of original signed texts, whereas the feasible forms were discussed with native signers of PJM who assessed these negatively prefixed signs as new forms. Thus, it can be stated confidently that the prefixation of the morpheme NEG- to base signs in PJM is a productive process of creating lexical opposites.

**Table 3:** Signs that can (left column) and cannot (right column) take the prefix NEG-.

NEG+signs	*NEG+signs
NEG+DOTYKAĆ ‘not touch’	*NEG+OGLĄDAĆ ‘not watch’
NEG+PÓJŚĆ ‘not go’	*NEG+NAPRAWIĆ ‘not repair’
NEG+ZAPOMNIEĆ ‘not forget’	*NEG+SZUKAĆ ‘not look for/not seek’
NEG+SPOTKAĆ ‘not meet”	*NEG+DOMYŚLAĆ-SIĘ ‘not guess’
NEG+UCZCIWY ‘dishonest’	*NEG+ROSNĄĆ ‘not grow’
NEG+PILNOWAĆ ‘not guard’ (variant 1)	*NEG+OBLICZYĆ ‘not calculate/not count’
NEG+PILNOWAĆ ‘not guard’ (variant 2)	*NEG+ZAMÓWIĆ ‘not order/not book’
NEG+PEWNY ‘not sure’	*NEG+WZIĄĆ ‘not take’
NEG+SKUTKOWAĆ ‘not take effect/not be effective’	*NEG+WPAŚĆ ‘drop in’
NEG+MUSIEĆ ‘not have to ‘	*NEG+ORGANIZOWAĆ ‘not organize’
NEG+WYPADAĆ ‘inappropriate’	*NEG+WEZWAĆ ‘not summon’
NEG+AKCEPTOWAĆ ‘not accept’	*NEG+PRZYZNAĆ ‘not admit’
NEG+TRAFIĆ ‘not hit’	*NEG+ŻAŁOWAĆ ‘not regret’
NEG+RÓWNY ‘uneven/unequal’	*NEG+SZCZERY ‘not sincere/ insincere’
NEG+PRZYZWYCZAJONY ‘not be accustomed’	*NEG+CZYSTY ‘not clean’
NEG+POWINIEN ‘should not’	*NEG+PROWADZIĆ ‘not lead somebody to’
NEG+SKOŃCZYĆ ‘not finish’	*NEG+BRONIĆ ‘not defend’
NEG+PRZYJĄĆ ‘not enroll’	*NEG+ZARZUCIĆ ‘not accuse’
NEG+PRZYJECHAĆ ‘not come/not arrive’ (variant 1)	*NEG+PRZYJECHAĆ ‘not come/not arrive’ (variant 2)

The analysis presented here involves noting the meaning of each sign and identifying the phonological sign parameters, such as handshape, orientation, movement and location. At the phonological level, the two groups (NEG+signs and *NEG+signs) point to constraints on the sequence of manner of articulation in prefixed signs.

The morpheme NEG- has an [arc convex] path movement [A], accompanied by a change in hand orientation. The first group of lexical items all have a path movement with the features straight (single), or restrained (double), and none of them involve a change in hand orientation. It is worth adding here that this group also includes signs that have a path movement with orientation internal movement resulting in an arc-like shape (e.g. ZAPOMNIEĆ ‘forget’ in PJM). This movement is, however, not specified with the [arc] feature. Such signs are characterized by branching orientation features that indicate rotation. The path movement is not specified at the underlying level at all, instead it is, by default, assigned [-arc], i.e., straight movement [31]. In contrast, the lexical items in the second group have other kinds of movement, such as fully circular repeated movement [C-rep] (OGLĄDAĆ ‘watch’, NAPRAWIĆ ‘repair’, SZUKAĆ ‘look for/seek’), arc concave (or convex) movement [A] (ZAMÓWIĆ ‘order/book’, WZIĄĆ ‘take’, WPAŚĆ ‘drop in’), secondary (without/with path) movement [Sec] (DOMYŚLAĆ-SIĘ ‘guess’, ROSNĄĆ ‘grow’, OBLICZYĆ ‘calculate/count’), or a change in hand orientation [OC] (ORGANIZOWAĆ ‘organize’, WEZWAĆ ‘summon’, PRZYZNAĆ ‘admit’). The contrast between these groups suggests that the process of prefixation involves a constraint on the sequences of movement and a constraint on the change of orientation, as given in (1) and (2).

(1) Movement Features Avoidance (MFA)
  *NEG-PREF ([A])/____([C-rep])
  *NEG-PREF ([A])/____([A])
  *NEG-PREF ([A])/____([Sec])

“A negative prefix containing an [A] is prohibited before bases with [C-rep], [A] and [Sec]”.

(2) Orientation Change Avoidance [OCA]
  *NEG-PREF ([OC])/____([OC])

“A negative prefix containing a [OC] is prohibited before bases with [OC]”.

The MFA and OCA restrictions concern bimorphemic and disyllabic (or tri-syllabic) prefixed signs. The MFA restriction means that if the prefix NEG- with the feature [A] is attached to a lexical item, the only acceptable sequence is arc convex movement + straight/restrained movement. It is not possible to have a sequence of convex arc movement + full circular (repeated) movement, arc movement + arc movement, arc movement + secondary (e.g. wiggling, bending) movement. In a given word, the sequence convex arc–straight/restrained is acceptable, but the sequences *arc–arc, *arc–full circular (repeated) or *arc–secondary are ruled out. The non-allowed sequence of the type *arc-arc in prefixed signs does not apply to those rare signs in the PJM lexicon that indeed use a true sequence of arc-arc movements but without any change in hand orientation (e.g. KONTYNUOWAĆ ‘continue’, REŻYSER ‘director’).

The OCA restriction does not accept any sequence of change in hand orientation + change in hand orientation. It is important to note that from the point of view of movement directionality, MFA and OCA apply to prefixed signs in which the lexical items have only unidirectional movement occurring either once (single) or repeatedly (double) or have only bidirectional (double) movement (in disyllabic signs).

**Fig 7:**
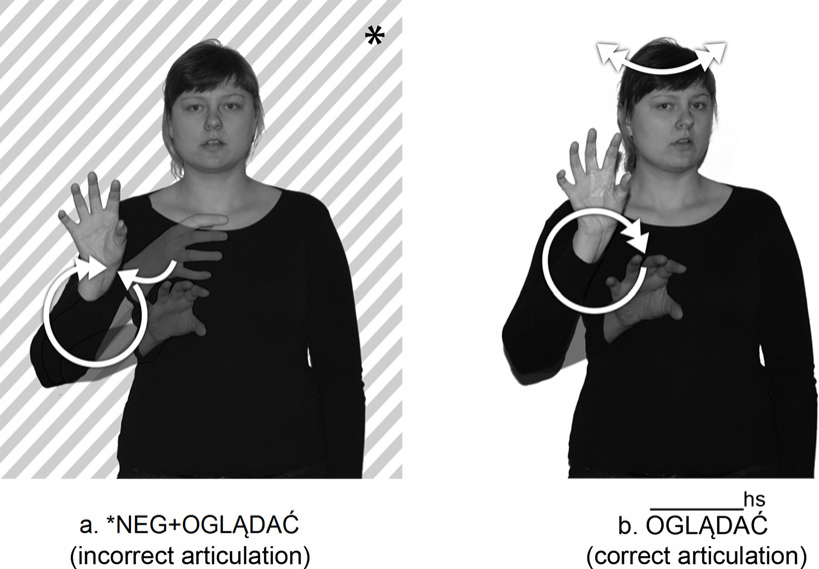
Examples of two signs. The individual in this figure has given written informed consent (as outlined in PLOS consent form) to publish these case details.

Fig 7, below, shows an example of two signs. The first is incorrect and the second is correctly formed from the standpoint of MFA.

The sign *NEG+OGLĄDAĆ ‘not watch’ (Fig 7A) contains the disallowed sequence of movements *convex arc + full circular (repeated). By contrast, the second sign OGLĄDAĆ ‘watch’ (Fig 7B) contains a permitted morphological process with a non-manual signal. This sign is accompanied by head shaking (hs) and means ‘not watch’. The negative non-manual element of head shaking forms part of the PJM grammar [33]. It may be added simultaneously to manual signs acting as predicates or to certain adjectives to which the marker NEG- cannot be attached. Similarly, the PJM prefixed signs can be optionally accompanied by head shaking.

**Fig 8:**
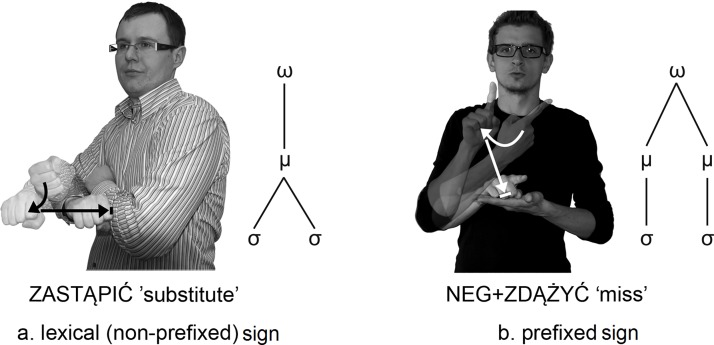
The comparison of two signs with identical sequences of movements: convex arc-straight. **Abbreviations**: ω, word; μ, morpheme; ơ, syllable. The two individuals in this figure have given written informed consent (as outlined in PLOS consent form) to publish these case details.

It is worth noting here, that MFA also takes place with some monomorphemic disyllabic signs in which the sequence of movements convex arc–straight is allowed. PJM signs of this kind include, for example, ZASTĄPIĆ ‘substitute’ (Fig 8A). The sequential restrictions on movements which function at the syllabic level rely on the fact that the reversed sequence of movements (*straight–convex (or concave) arc.) is not allowed. In PJM, an arc movement may not occur after a straight movement. An arced movement frequently occurs before a straight movement but not after. The articulation of monomorphemic disyllabic signs with the sequence *straight–arc would thus seem to be in violation of phonotactic rules.

In summary, MFA occurs in PJM in connection with prefixed signs and monomorphemic disyllabic signs. Fig 8 shows two signs with identical convex arc–straight sequences of movements.

There are still some important issues that should be addressed. The two constraints, MFA and OCA, seem to be relevant to three assumptions, two of which argue for Sandler’s [31,39] position based on ASL and the third supports Sandler’s [35,39] statement based on studies of ISL. The first argument concerns the circling movement [C-rep] which is considered to consist of a sequence of arcs with different values of concavity. Therefore, the movement feature [C-rep] has the underlying form of an arc with an epenthetic arc of the opposite shape: if the underlying arc is concave, the epenthetic one is convex and vice versa [31]. The second argument says that handshape and orientation changes are both subsumed by an inventory of internal movement [39]. The third argument is that straight path movement is a default (unspecified) type [35], and other kinds of movement, (such as arcs, tense, doubled, that is, restrained) are specified (non-default) in the lexicon [39]. With the latter two assumptions, since the PJM prefix NEG- typically contains both an arc movement and an orientation change, the constraint seems, informally, to be:

(3) Specified Movement Constraint (SMC) on prefixed prosodic words in PJM:

“A sequence of two specified movements prevents syllable reduction in prefixed signs”.

The SMC may result from the specificity of the movement which is separate, potentially independent and established, whereas default movements have no any such features. This restriction has been observed in the data obtained from the PJM corpus; some of the 15 subjects used some of the prefixed signs alternately as monosyllabic words (e.g. NEG+ZGADZAĆ-SIĘ ‘disagree’, NEG+DAĆ ‘not give’, NEG+BĘDZIE ‘will not’, NEG+MUSIEĆ ‘not have to’). These prefixed signs have a sequence of specified movement (arc) + default movement (straight). S4 Fig and S29 Video show the phenomenon of syllable shortening in the prefixed sign NEG+ZGADZAĆ-SIĘ ‘disagree’, which formally has a two-syllable structure, or a sequence of two movements, the first of which is specified, the second is default (Fig 4C). This phonological process, that reflects the tendency toward monosyllabicity in prefixed signs, occurs when the first specified movement is lost and the change of hand orientation is not violated. This is linked with the second default movement with the feature [straight]. Therefore, the first syllable is lost and NEG+ZGADZAĆ-SIĘ becomes a monosyllabic sign, in which internal movement occurs together with path movement, and so path and orientation change simultaneously as one type of movement in lexical signs. As far as prefixed signs that contain sequences of two specified (arc+restrained) movements (e.g. NEG+POTRZEBNY 'unnecessary'; S7 Video, NEG+ROZUMIEĆ 'not understand'; S9 Video) are concerned, they have not been observed to undergo syllable shortening, which would seem to confirm the SMC on prefixed prosodic words in PJM.

In the group of *NEG+signs (Table 3), there are also signs that have the feature [contact] in the location segment (creating an initial contact; SZCZERY ‘sincere’), on the movement segment (creating a brushing movement: PROWADZIĆ ‘lead somebody to’), and on all segments (for continuous contact; CZYSTY ‘clean’). Additionally, there are signs that have only one location with the [contact] feature (ŻAŁOWAĆ ‘regret’). Apparently, the prefix NEG- cannot be combined with the signs mentioned, indicating the occurrence of the next constraint on this negator, as given in (4).

(4) Contact-initial Avoidance (C-iA)
  *NEG-PREF ([S])/____([C-i])

“A negative prefix containing a spatial location [S] is prohibited before contact-initial [C-i] bases”.

The C-iA constraint means that the prefix NEG- with a spatial location [S] (not on the body) cannot be attached to lexical signs which include the feature [contact] in the initial location segment, on the movement segment, or on all segments. Hence, it is not possible to combine the final spatial location of the marker NEG- with an initial contact location [C-i] of the base sign. However, the negative prefix can be added to signs with contact on the final location (NEG+ZGADZAĆ-SIĘ ‘disagree’). This shows that there is a non-contact rule in the process of prefixation. The C-iA constraint confirms the claim that locations are important phonological units of sign structure [31]. What is more, the [contact] feature plays an active role in sign language phonology [40]. This also shows that the structure of most ASL signs is sequential, as it distinguishes between initial and final locations [1,31].

It is important to note that the group of *NEG+signs (Table 3) also contains signs that have straight path movement, but they simultaneously co-occur with mouth actions from PJM. Research on the behavior of non-manual signals in PJM has shown that the mouth as a non-manual articulator has the widest potential range of uses due to the greater number of mouth components possible in comparison with other non-manual articulators [30]. What is more, two kinds of mouth actions have been attested in the articulation of signs: *mouth gesture* and *mouthing* [41]. The mouth gestures [MG] typical for PJM have nothing in common with the oral articulation of spoken Polish. Mouthing [M], on the other hand, refers to the voiceless articulation of a complete or partially spoken word during the articulation of a sign. For example, the PJM sign BRONIĆ ‘defend’ has a mouth component [airstream], and the sign ZARZUCIĆ ‘accuse’ has the bisegmental components [bilabial, open]. These mouth components are typical of PJM. Since the prefix NEG- is derived from the #NIE ‘no/not’ (borrowing from the Polish language), it co-occurs with the mouth pattern [bisegmental components (tongue, open)] related to spoken Polish. Despite the fact that these mouthing features of the morpheme NEG- can be weak (and can in fact be lost) during the process of prefixation, it cannot be attached to lexical items with mouth gestures belonging to PJM. Hence, there is a following constraint, given in (5):

(5) Mouth Gesture Avoidance (MGA)
  *NEG-PREF ([M])/____([MG])

“A negative prefix containing a [M] is prohibited before bases with [MG]”.

The MGA restraint accepts the mouth pattern sequence: mouthing–mouthing. It is not possible to have a sequence: *mouthing–mouth gesture. For example, the prefix NEG- can be attached to the PJM sign MUSIEĆ ‘must’ containing a sequence of two mouth components [bilabial] and [round] which come from the two initial sound [m] and [u] in the Polish word ‘musieć’ (‘must’). However, the marker NEG- is not allowed to be combined with the lexical phrase ZARZUCIĆ ‘accuse’ because it has bisegmental components [bilabial, open] which belong natively to PJM. As the two groups of signs show (Table 3), there are two stylistic variants of sign PRZYJECHAĆ ‘come/arrive’. The first has mouthing components and so can be combined with the prefix NEG-; The second contains a mouth gesture component which precludes the negative marker. The tendency for Deaf PJM users to use mouth actions related to spoken words, especially those being directly negated can be considered to be an example of the influence of Polish on PJM.

Four of these rules (MFA, OCA, C-iA and MGA) involve some notion of ‘avoidance’. It seems possible to collapse the three rules (MFA, OCA and C-iA) into one broad rule named ‘The Principle of Uneconomical Connection Parameter Avoidance’ (movement, hand orientation, location). This refers to the avoidance of certain phonological combinations that make it difficult to recognize prefixed signs, which is associated with language economy. This confirms Zipf’s [42] principle of least effort in that the distribution of word usage was due to tendency to communicate efficiently with the least effort possible. At this stage, this remains a working hypothesis. To confirm it, further corpus-based research into the broader contexts, in which such forms are used in accordance with the MFA, OCA and C-iA rules is necessary.

The fourth constraint (MGA) is different in nature. It could be described as a principle of avoiding hybridization as the process of a non-native component blending with a native component. This rule seems to involve cognitive action of the body in PJM: PJM mouth gestures avoid combination with non-native mouthing components belonging to spoken Polish.

Aronoff et al. [4] noted a phonological constraint on the ASL negative suffix -ZERO whose structure consists of a one-handed form: it can be attached only to one-handed stems and not to two-handed base signs. Unlike this effect, it was found that in PJM the base sign determines whether the one-handed prefix NEG- is one- or two-handed. It is described as the handedness rule for NEG-.

(6) Handedness Rule (HR):

“A one-handed prefix NEG- becomes two-handed when affixed to two-handed bases”.

Thus, if the base sign is one-handed, the prefix will be one-handed as well, like the prefixed sign NEG+ROZUMIEĆ ‘not understand’ (Fig 5B). As regards two-handed base signs, it is important to note differential effects of combining the prefix NEG- with h2-S (symmetry) signs, in which both hands move symmetrically (h2 performs the same phonological role as h1) and h2-P (place) signs in which only h1 moves and h2 performs the phonological function of place of articulation.

**Fig 9:**
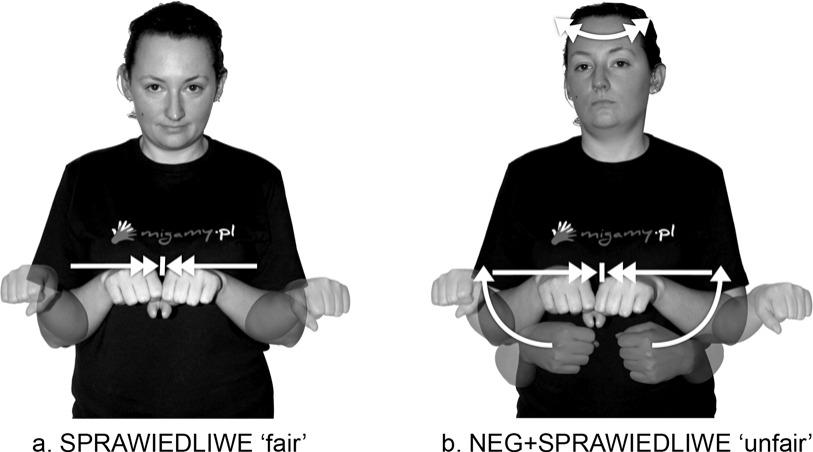
The PJM signs SPRAWIEDLIWE ‘fair’ and NEG+SPRAWIEDLIWE ‘unfair’. The individual in this figure has given written informed consent (as outlined in PLOS consent form) to publish these case details.

As shown in Figs 4C and 5C, the h2-P type signs are formed in such a way that, while the prefix NEG- is being articulated with the dominant hand (h1), the non-dominant hand (h2) is obligatorily placed in the location of the second sign in front of the signer’s body. However, the h2-S type signs are articulated differently from the h2-P type signs. The h2-S signs require the prefix NEG- to be two-handed: Both hands of the prefix must be specified for the same handshape, the same movement, and the same orientation; these signs must be articulated symmetrically and identically, in accordance with Battison’s Symmetry Condition [27,28]. An example of this is the prefixed sign NEG+SPRAWIEDLIWE ‘unfair’, in which both hands perform the same phonological action (Fig 9B, see also other h2-S signs with the NEG- prefix, for example, in S5, S20 and S21 Videos).

Allomorphic variation of the prefix then is determined by the phonological structure of the stem and is characteristic of affixes. In ISL, the same happens with the negative suffix NOT-EXIST, whose allomorphic variation (one-handed vs. two-handed forms) is determined by the phonological structure of the base [43]. In other words, there is a handedness constraint on the ASL suffix–ZERO and the ISL suffix NOT-EXIST. The one-handed suffix–ZERO is prohibited after bimanual bases and the two-handed suffix NOT-EXIST must become one-handed when affixed to one-handed bases. This rule in PJM however does not constrain the one-handed prefix NEG- and may be adapted phonologically to two-handed lexical morphemes becoming two-handed in the process. The fact that this possibility is not available to the suffix–ZERO in ASL may perhaps result from its phonological status.

Finally, it is worth noting that there is also a morphological constraint on the negative prefix bases, in that NEG- can only be attached to plain verbs (including adjectives), and not to agreement verbs, spatial verbs or classifier predicates. Some examples are presented in S3 Table: It is possible to express the meaning of negation with agreement verbs or classifier predicates to which the prefix NEG- cannot apply. These verbs can be accompanied by a non-manual element of negation in the form of a head shake. Alternatively, the negative expression #NIE can follow agreement verbs and classifier predicates (see S3 Table). The impossibility of attaching NEG- to agreement and spatial verbs seems to be associated with the characterization of the three verb classes: plain verbs, agreement verbs and spatial verbs. As Padden [44,45] noted, these classes differ from each other with respect to the properties of the arguments which they encode. Plain verbs, which have one verb form, do not encode referential properties of arguments. Unlike plain verbs, agreement verbs encode person and number features of their subject and object arguments. The complexity of the syntactic role of agreement verbs seems to prevent the prefix NEG- from being attached to these verbs. Since plain verbs do not encode any grammatical features of their arguments, the NEG- can be combined with these verbs with no loss in meaning. An attempt to attach the prefix NEG- to agreement verbs could distort the encoding of the relevant grammatical features, which would make it difficult to identify the arguments expressed, as well as their person and number features. The same restriction applies to spatial verbs (verbs of motion with classifier constructions). They encode the locative roles of arguments. Therefore, attaching the prefix NEG- to these verbs may block the denotation of motion and location in space.

### Comparing constraints on compounding and prefixation

The previously discussed constraints on negative prefixation provide another argument that the morpheme NEG- is a prefix rather than the first element of a sequential compound or an independent sign. Still, a comparison of constraints on sequential compounding and prefixation should be made in order to identify possible similarities or differences between them. This would help improve understanding of the nature of constraints at the phonological and morphological levels.

In comparing these constraints, the morphological and phonological rules distinguished by Liddell [46] and Liddell & Johnson [47], which are applied in the sequential compounding process in ASL when new compound signs are formed from two sign roots (Tables 4 and 5), are taken into account. It should be borne in mind that the compound phenomena described here refer to lexicalized compounds but not to all productively formed novel compounds. At any rate, these rules are also applied in the formation of sequential compounds in such sign languages as British Sign Language [48], Indopakistan Sign Language [49], New Zealand Sign Language [50], Polish Sign Language [51], Australian Sign Language [8], Israeli Sign Language [9] and Irish Sign Language [10].

**Table 4:** Comparison of morphological constraints on sequential compounding and prefixation.

Morphological rules	
COMPOUNDING	PREFIXATION
*The first contact rule*. The hold segment of one of the two signs remains in contact with the body or with the second hand. During the process of linking signs, the contact of the holding segment of one of the signs is maintained.	There is a non-contact rule that has a different effect than “the first contact rule” used to form compounds. For prefixation, this rule disallows attaching the negative prefix NEG- to stems, whose (first) location segment includes contact on the body or the other hand (contact feature). As previously mentioned, this effect is called contact-initial avoidance (CiA).
*The simple sequence rule*. When sign compounds are formed, internal movement or repetition of movement is eliminated.	No simple sequence rule exists for prefixation. Instead, when prefixed signs are formed in PJM, or repetition of movement (with the feature ‘restrained’) of the lexical sign is maintained. In compounding, internal movement (e.g. the wiggling of the fingers) of one of the two signs is eliminated, while the negative prefix NEG- is avoided with stems having internal movement (e.g. wiggling, bending), which occurs with the spatial location segment (e.g. *NEG+DOMYŚLAĆ-SIĘ ‘not guess’).
*The weak hand anticipation rule*. When two signs are combined to form a compound, it often happens that the signer’s weak hand anticipates the second sign in the compound.	*This rule also applies to affixation*. When the NEG- morpheme and h2-P sign (only h1 moves, h2 performs the phonological role of place of articulation) are combined to form a prefixed sign, a h2 anticipates the base sign. An example of h1 anticipation in prefixation can be seen in the prefixed sign NEG+ZDĄŻYĆ ‘miss’: the h2 appears with the “B” handshape of the sign ZDĄŻYĆ ‘make it’ at the same time that the h1 (active hand) is articulating the prefix NEG- (see Fig 4C).

**Table 5:** Comparison of phonological constraints on sequential compounding and prefixation.

Phonological rules	
COMPOUNDING	PREFIXATION
*Movement epenthesis* A movement segment is added between the last segment of one sign and the first segment of the next sign.	There is no movement epenthesis in prefixation which never involves adding a movement segment between the last segment of the prefix NEG- and the first segment of the stem. This kind of movement cannot be added between the final location of the NEG- and the first location of lexical item in which contact-initial avoidance (C-iA) appears.
*Hold (location)deletion* When two signs come together to form a compound, the noncontact holds (locations) between movements are eliminated.	When two morphemes come together to form a prefixed sign, the noncontact location segments between movements are not eliminated. However, there is a different phonological effect with prefixation: the last noncontact location of the prefix NEG- and the first noncontact location of the lexical item are combined into one common location with a feature which is used to characterize the first location of second sign. This is a noncontact rule.
*Assimilation* A segment takes on the characteristics of another segment near it.	*This rule is also true for prefixation*. The PJM prefixed signs undergo handshape and location assimilation (also orientation assimilation in some prefixed signs), a process that also occurs in compounds but does not occur across independent signs. The handshape of the prefix NEG- is assimilated to the handshape of the lexical item, to which the prefix is attached. Also the location of NEG- is partly assimilated to the location of the stem.

The description of the morphological and phonological constraints on the composition and negative prefixation (Tables 4 and 5) point to the similarity of rules concerning anticipation and assimilation. However, as it is turns out, there are more differences resulting from the fact that the prefix NEG- is more phonologically “reduced” compared to a morpheme within a compound since spatial location and arc movement, with which the negator NEG- works, prohibit it from attaching to bases with initial contact. This non-contact rule is closely related to a restriction that disallows movement epenthesis and which specifically enables the formation of a compound using two free morphemes. The MFA constraint, which occurs with negative prefixation, disallows the elimination of segments in signs, to which prefixes are added. An expression of this is the rejection of bases with internal movement or complex combinations of movements that have different features. This kind of affixation follows the rule that prefixed signs have a structure of at least two-syllables. This does not apply, however, in the case of sequential compounding; sign compounds tend to have single syllables [1].

A comparison of negative prefixation and compounding shows important differences. It was found that there is no epenthesis of movement between the final segment of the prefix NEG- and the first segment of the stem. In other words, in PJM, the prefixation of NEG- disallows the use of epenthetic movement, favoring instead the direct linear combination of NEG- with lexical items. This type of linear combination seems to be governed by morphophonological constraints, where syllables are not reduced; complex signs with the prefix NEG- have at least a disyllabic structure despite the possible syllable shortening of some prefixed signs. Thus, morphophonological constraints on affixation seem to emerge when the process of affixation results in the direct adjacency of morphemes, with no epenthetic movement between them.

## Conclusion and Implications

An analysis of affixation in PJM attests to the fact that there is a negative prefix NEG- which can be attached to many base signs. It suggests that the typological model of irregular negatives in sign languages should be revised since this model states that “all known cases in sign languages involve a negative morpheme following the stem” (see Results in [5]). As in other documented sign languages, PJM uses suffixed negators as well. The existence of constraints on the affixation of the prefix NEG- seems to be sufficient for explaining the phenomenon. There are phonological restrictions on the stems, with which the negative morpheme NEG- can combine. This may well be language-specific, for example movement must not be complex within a sign and there can only be one orientation change per sign. Moreover, phonological constraints on morphological processes have been described for other sign languages. In German Sign Language, for example, the plural reduplication of nouns is impossible when the noun has complex movement or is body-anchored [52].

It is necessary to conduct further comparative research into restrictions on prefixation and suffixation both in PJM and other sign languages (e.g. ASL, ISL) to explore more deeply the function of epenthetic movement in grammaticalization. This would make it possible to illuminate how differences between sign languages may arise. In that case, it may be supposed that in many suffixation processes in sign languages (including PJM), the linear combination of two morphemes involves the insertion of epenthetic movement. This epenthetic movement makes it possible to combine any two morphemes, regardless of their phonological properties. Thus, there would not necessarily be strict morphophonological constraints on the process of suffixation in sign languages when epenthetic movement is involved. Nevertheless, in some cases of suffixation, there are rules under which epenthetic movement disappears and a reduction of syllables occurs. A good example is a PJM suffix–NIE-MA ‘not exist’ which can be used with nouns. At one time, this suffix was attached to the base and epenthetic movement was added between them, but now this parameter is disappearing from suffixed stems. This phenomenon indicates the development of free functional elements from lexical stems as type-1 grammaticalization and the development of affixes from lexical or free functional elements as type-2 grammaticalization [52]. Hence, it can be hypothesized that in the first stage of grammaticalization, the suffix–NIE-MA ‘not exist’ functioned as a free element that could be called a “proto-suffix” in that it did not have the full status of an affix. In the second stage of grammaticalization, however,–NIE-MA ‘not exist’ became a full suffix which cannot function independently and must therefore be connected with the base when there is no epenthetic movement within suffixed sign. It seems that a deeper analysis of these grammaticalization paths might make it possible to describe the source of constraints on affixation of the derivational morphemes occurring in sign languages. This is the goal of future research.

Not only in spoken but also in sign languages the possibility of violating constraints in specific phonological contexts has been researched [1, 53, 54]. It would be therefore worthwhile to more profoundly examine, based on corpus utterances, whether the conditions outlined in this work for the formation of prefixed signs could be violated.

It is also worth raising the issue of the previously described possibility of shortening some prefixed signs to one syllable. As is already known, PJM prefixed signs with NEG- are bimorphemic and have at least a dissyllabic structure (the prefix has only one syllable and the base may have one or two syllables) and so they could not be less than one syllable long. As it turns out, however, in the case of shortening some prefixed signs to one syllable, it is possible to eliminate the movement segment with the [arc] feature belonging to the prefix NEG-, while maintaining the change of orientation that results from the internal movement. It can be assumed that PJM dissyllabic prefixed signs have a strong-normal stress pattern, “where stress is characterized as increased muscle tension and increased speed” (see Results in [1]). These prefixed signs could be described as being stressed on the first syllable, that is on the NEG- prefix, since this morpheme involves an orientation change combined with path movement, thus indicating a certain degree of sonority as “visual perceptual salience” (see Results in [53]). That is, this morpheme is articulated with greater force or intensity, or, in other words, it has dynamic stress. And the second morpheme, as a one-syllable base, to which the prefix NEG- is attached, seems to retain normal stress. However when the monosyllabic shortening of the prefixed sign with two sequential movements (specified+default) occurs, the stress is “transferred” to the default path movement belonging to the lexical base. It is worth noting that a monosyllabic sign such as NEG+ZGADZAĆ ‘disagree’ (with path movement and orientation change) is more sonorous than the monosyllabic sign ZGADZAĆ-SIĘ ‘agree’ (with only single path movement). It is necessary to conduct further research into the process of syllable shortening prefixed signs in PJM. A comparison of PJM negative prefixed signs with other two syllable signs having different stress patterns, which have been described by some ASL researchers [55, 56, 57], could provide a cross-linguistic perspective that could improve understanding of the signed syllable.

Indeed, the initial results of the present research on negative prefixation in PJM indicate the possibility of syllabic shortening some prefixed signs as well as a "conspiracy of monosyllabicity", in line with Sandler [31, 58] and Brentari [53]. This requires, however, deeper corpus-based research in order to verify the hypothesis concerning the SMC on prefixed prosodic words in PJM.

The present study focused only on the phonological and morphological constraints on the application of the prefix NEG- to plain verbs and adjectives. The restrictions which seem to exist with negatively prefixed adjectives in PJM are predominantly semantic in nature. Future research is therefore necessary to determine exactly what is involved in this kind of constraint, which is one of the factors limiting morphological productivity in PJM. Also sign phonology is a factor in the distribution of allomorphs. In this paper, it was mentioned that there are phonological processes in negative prefixation such as assimilation. Therefore, the effect they have on the shapes of morpheme NEG- should be precisely described in order to identify the number of allomorphic variations of this negator.

In closing, this study of the prefix NEG- has shown the occurrence of morphophonological constraints on syllable sequences in prefixed signs which indicate that the movement and change of orientation must not be too similar in each syllable. Deeper analysis of the constraints on the negation prefix NEG- in PJM would allow a more detailed description of the patterns found in the process of grammaticalization in the visual-gestural modality.

## Supporting Information

S1 Fig The prefixed sign NEG+ZGADZAĆ-SIĘ ‘disagree’ used by a Deaf signer. The individual in this figure has given written informed consent (as outlined in PLOS consent form) to publish these case details.
(TIFF)

S2 Fig The sequence of movements in the prefixed sign NEG+ROZUMIEĆ ‘not understand’ (view from above). A sequence of two different motions: arc movement (1) + restrained movement (2).
(TIFF)

S3 Fig Occurrence rate for various prefixed signs (in percentage). (TIF)

S4 Fig Syllable reduction in the prefixed sign NEG+ZGADZAĆ-SIĘ ‘disagree’ used by a Deaf signer. The individual in this figure has given written informed consent (as outlined in PLOS consent form) to publish these case details.
(TIF)

S1 Table Eight criteria for the lexicalization of fingerspelled loan signs according to Battison [27, 28]. (TIF)

S2 Table Number (*n*) of different prefixed signs in a corpus collected from 15 Deaf informants. (TIF)

S3 Table Comparison of two categories of sign phrases. hs, non-manual element of negation in the form of a head shake.
(TIF)

S1 Text PJM rhetorical question with the negator # NIE ‘no/not’. In the first part of the sentence shown above in PJM (with Polish words in capital letters standing for PJM signs). There is a rhetorical question (rh) which precedes the final negative element in a stylistically marked way. In this question there are non-manual elements similar to those that occur in yes/no questions, such as raised eyebrows and wide open eyes. However, by the sign TAK-DOBRZE ‘very good’, there is a change in the non-manual elements which sententially apply to the negative expression #NIE. Then the eyebrows are lowered, the nose slightly wrinkles and, most importantly, there is an obligatory head shake (neg).
(TIF)

S1 Video Prefixed sign NEG+ZDĄŻYĆ ‘miss’ at 0.3. The individual in this video has given written informed consent (as outlined in PLOS consent form) to publish these case details.
(MP4)

S2 Video Prefixed sign NEG+ZGADZAĆ-SIĘ ‘disagree’ at 0.2. The individual in this video has given written informed consent (as outlined in PLOS consent form) to publish these case details.
(MP4)

S3 Video Prefixed sign NEG+DAĆ ‘not give’ at 0.1 and 0.3. The individual in this video has given written informed consent (as outlined in PLOS consent form) to publish these case details.
(MP4)

S4 Video Prefixed sign NEG+ŻYĆ ‘not alive’ at 0.5. The individual in this video has given written informed consent (as outlined in PLOS consent form) to publish these case details.
(MP4)

S5 Video Prefixed sign NEG+WINNY ‘not guilty/innocent’ at 0.4. The individual in this video has given written informed consent (as outlined in PLOS consent form) to publish these case details.
(MP4)

S6 Video Prefixed sign NEG+WYGODNY ‘uncomfortable’ at 0.2. The individual in this video has given written informed consent (as outlined in PLOS consent form) to publish these case details.
(MP4)

S7 Video Prefixed sign NEG+POTRZEBNY ‘unnecessary’ at 0.1. The individual in this video has given written informed consent (as outlined in PLOS consent form) to publish these case details.
(MP4)

S8 Video Prefixed sign NEG+SYMPATYCZNY ‘unlikeable’ at 0.5 and 0.7. The individual in this video has given written informed consent (as outlined in PLOS consent form) to publish these case details.
(MP4)

S9 Video Prefixed sign NEG+ROZUMIEĆ ‘not understand’ at 0.1. The individual in this video has given written informed consent (as outlined in PLOS consent form) to publish these case details.
(MP4)

S10 Video Prefixed sign NEG+DOTYKAĆ ‘not touch’ at 0.3. The individual in this video has given written informed consent (as outlined in PLOS consent form) to publish these case details.
(MP4)

S11 Video Prefixed sign NEG+PÓJŚĆ ‘not go’ at 0.1 and 0.3. The individual in this video has given written informed consent (as outlined in PLOS consent form) to publish these case details.
(MP4)

S12 Video Prefixed sign NEG+ZAPOMNIEĆ ‘not forget’ at 0.2. The individual in this video has given written informed consent (as outlined in PLOS consent form) to publish these case details.
(MP4)

S13 Video Prefixed sign NEG+SPOTKAĆ ‘not meet’ at 0.3. The individual in this video has given written informed consent (as outlined in PLOS consent form) to publish these case details.
(MP4)

S14 Video Prefixed sign NEG+UCZCIWY ‘dishonest’ at 0.2. The individual in this video has given written informed consent (as outlined in PLOS consent form) to publish these case details.
(MP4)

S15 Video Prefixed sign NEG+PILNOWAĆ ‘not guard’ (variant 1) at 0.3. The individual in this video has given written informed consent (as outlined in PLOS consent form) to publish these case details.
(MP4)

S16 Video Prefixed sign NEG+PILNOWAĆ ‘not guard’ (variant 2) at 0.1. The individual in this video has given written informed consent (as outlined in PLOS consent form) to publish these case details.
(MP4)

S17 Video Prefixed sign NEG+PEWNY ‘not sure’ at 0.6. The individual in this video has given written informed consent (as outlined in PLOS consent form) to publish these case details.
(MP4)

S18 Video Prefixed sign NEG+SKUTKOWAĆ ‘not take effect/not be effective’ at 0.6. The individual in this video has given written informed consent (as outlined in PLOS consent form) to publish these case details.
(MP4)

S19 Video Prefixed sign NEG+MUSIEĆ ‘not have to’ at 0.2 and 0.5. The individual in this video has given written informed consent (as outlined in PLOS consent form) to publish these case details.
(MP4)

S20 Video Prefixed sign NEG+WYPADAĆ ‘inappropriate’ at 0.1 and 0.5. The individual in this video has given written informed consent (as outlined in PLOS consent form) to publish these case details.
(MP4)

S21 Video Prefixed sign NEG+AKCEPTOWAĆ ‘not accept’ at 0.1. The individual in this video has given written informed consent (as outlined in PLOS consent form) to publish these case details.
(MP4)

S22 Video Prefixed sign NEG+TRAFIĆ ‘not hit’ at 0.4. The individual in this video has given written informed consent (as outlined in PLOS consent form) to publish these case details.
(MP4)

S23 Video Prefixed sign NEG+RÓWNY ‘uneven/unequal’ at 0.5. The individual in this video has given written informed consent (as outlined in PLOS consent form) to publish these case details.
(MP4)

S24 Video Prefixed sign NEG+PRZYZWYCZAJONY ‘not be accustomed’ at 0.5. The individual in this video has given written informed consent (as outlined in PLOS consent form) to publish these case details.
(MP4)

S25 Video Prefixed sign NEG+POWINIEN ‘should not’ at 0.3. The individual in this video has given written informed consent (as outlined in PLOS consent form) to publish these case details.
(MP4)

S26 Video Prefixed sign NEG+SKOŃCZYĆ ‘not finish’ at 0.1. The individual in this video has given written informed consent (as outlined in PLOS consent form) to publish these case details.
(MP4)

S27 Video Prefixed sign NEG+PRZYJĄĆ ‘not enroll’ at 0.2. The individual in this video has given written informed consent (as outlined in PLOS consent form) to publish these case details.
(MP4)

S28 Video Prefixed sign NEG+PRZYJECHAĆ ‘not come/not arrive’ at 0.6. The individual in this video has given written informed consent (as outlined in PLOS consent form) to publish these case details.
(MP4)

S29 Video Syllable reduction in the prefixed sign NEG+ZGADZAĆ-SIĘ ‘disagree’ at 0.1. The individual in this video has given written informed consent (as outlined in PLOS consent form) to publish these case details.
(MP4)
