# Supplementary figures and images for: Constraints on Negative Prefixation in Polish Sign Language
Source: PLoS One. 2015 Nov 30;10(11):e0143574. doi: 10.1371/journal.pone.0143574 (PMC4664272; doi:10.1371/journal.pone.0143574)

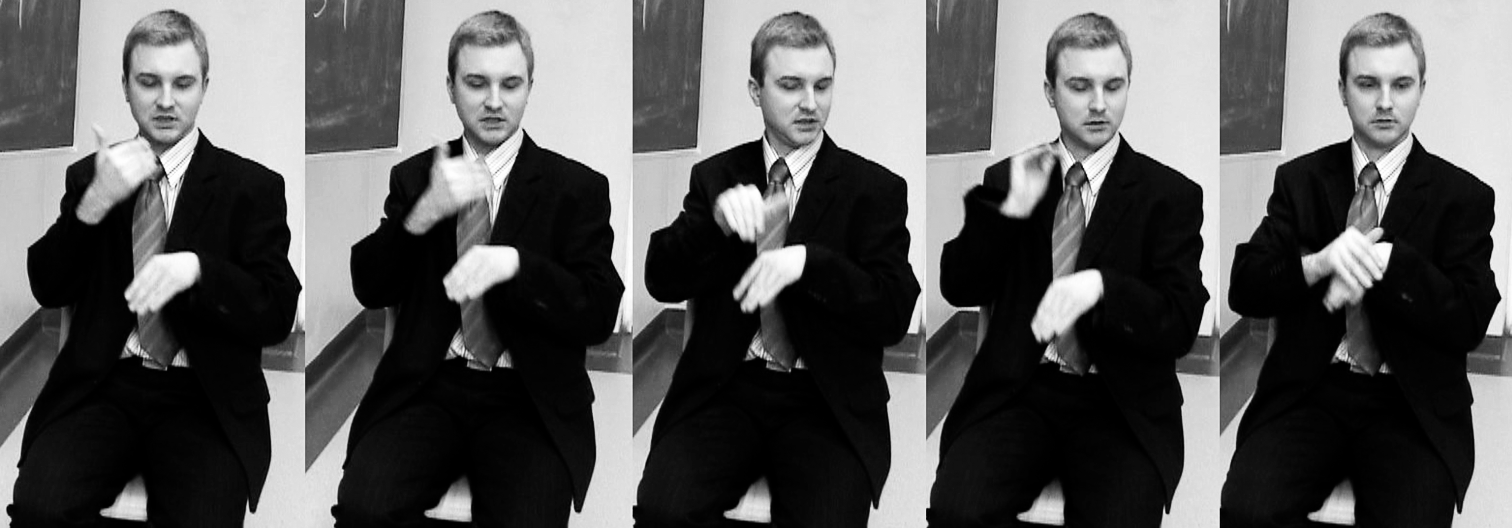

Supplement: S1 Fig — The individual in this figure has given written informed consent (as outlined in PLOS consent form) to publish these case details. (TIFF) [file pone.0143574.s001.tiff]

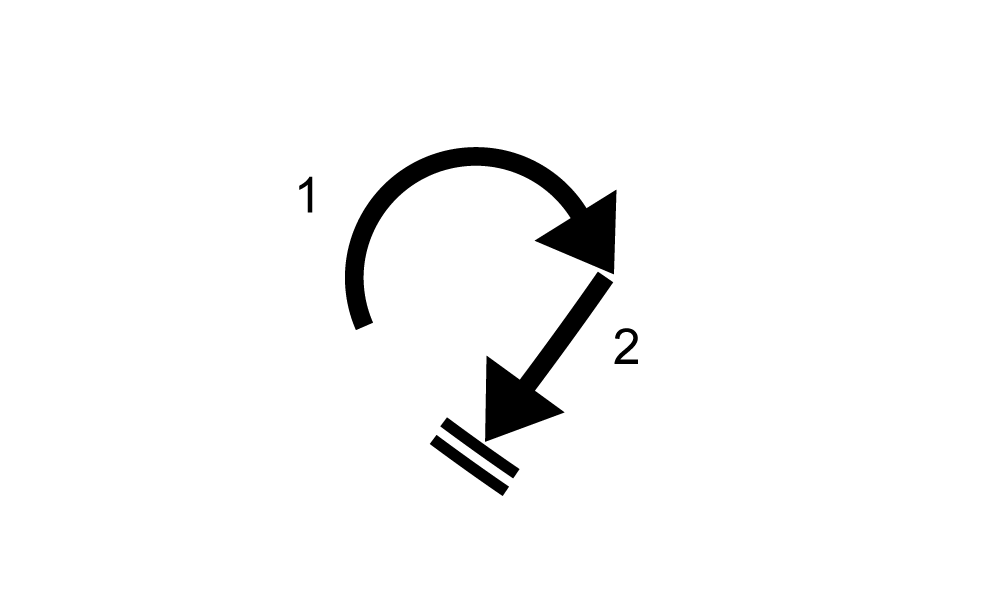

Supplement: S2 Fig — A sequence of two different motions: arc movement (1) + restrained movement (2). (TIFF) [file pone.0143574.s002.tiff]

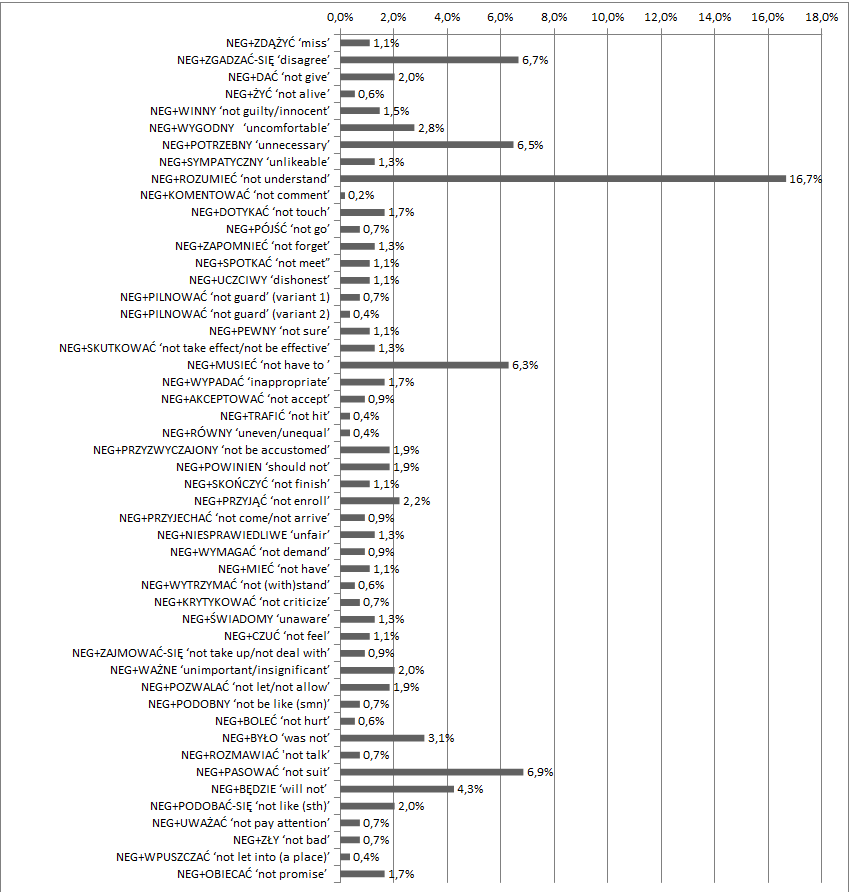

Supplement: S3 Fig — (TIF) [file pone.0143574.s003.tif]

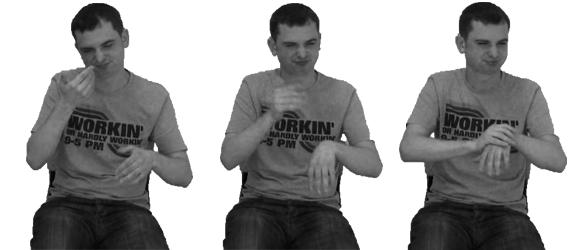

Supplement: S4 Fig — The individual in this figure has given written informed consent (as outlined in PLOS consent form) to publish these case details. (TIF) [file pone.0143574.s004.tif]

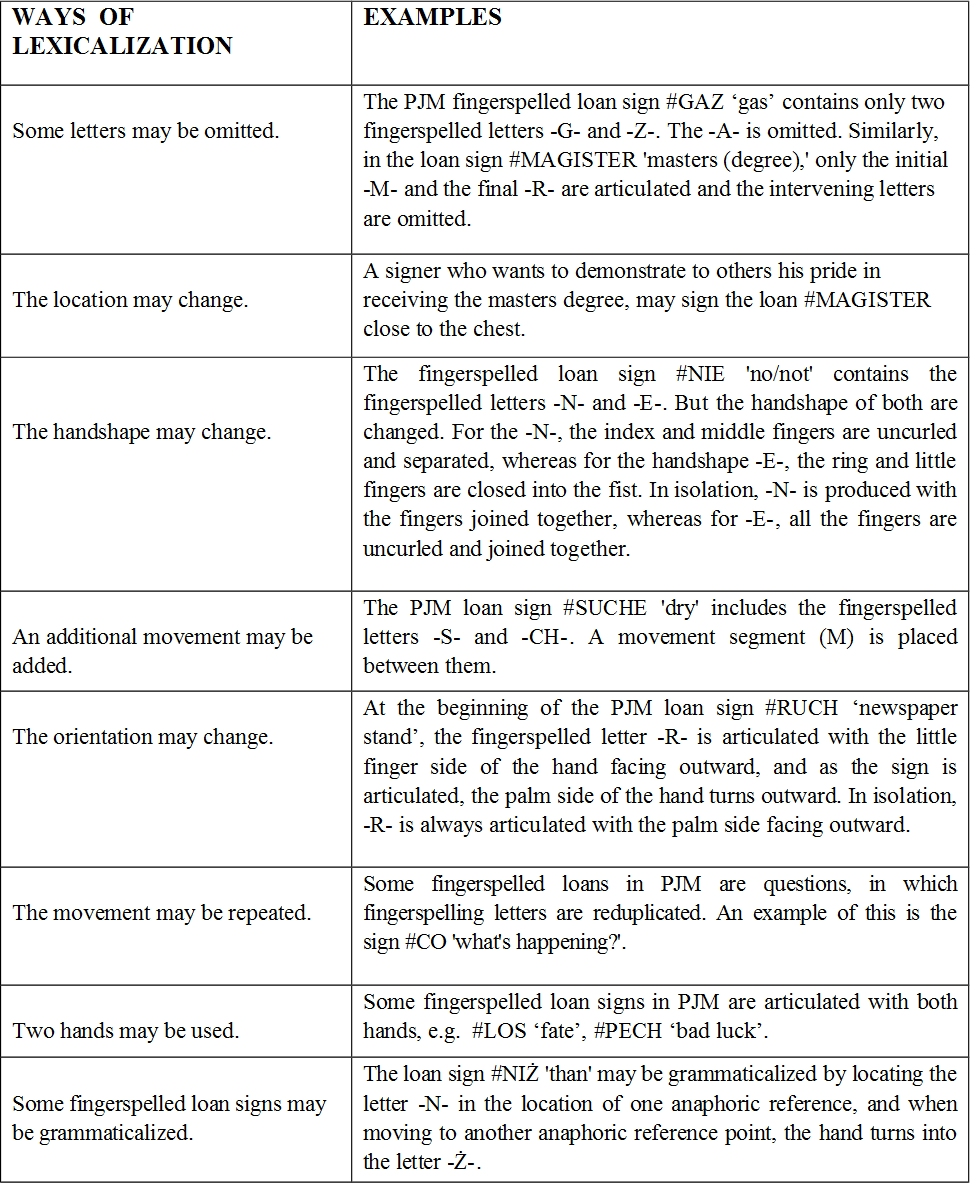

Supplement: S1 Table — (TIF) [file pone.0143574.s005.tif]

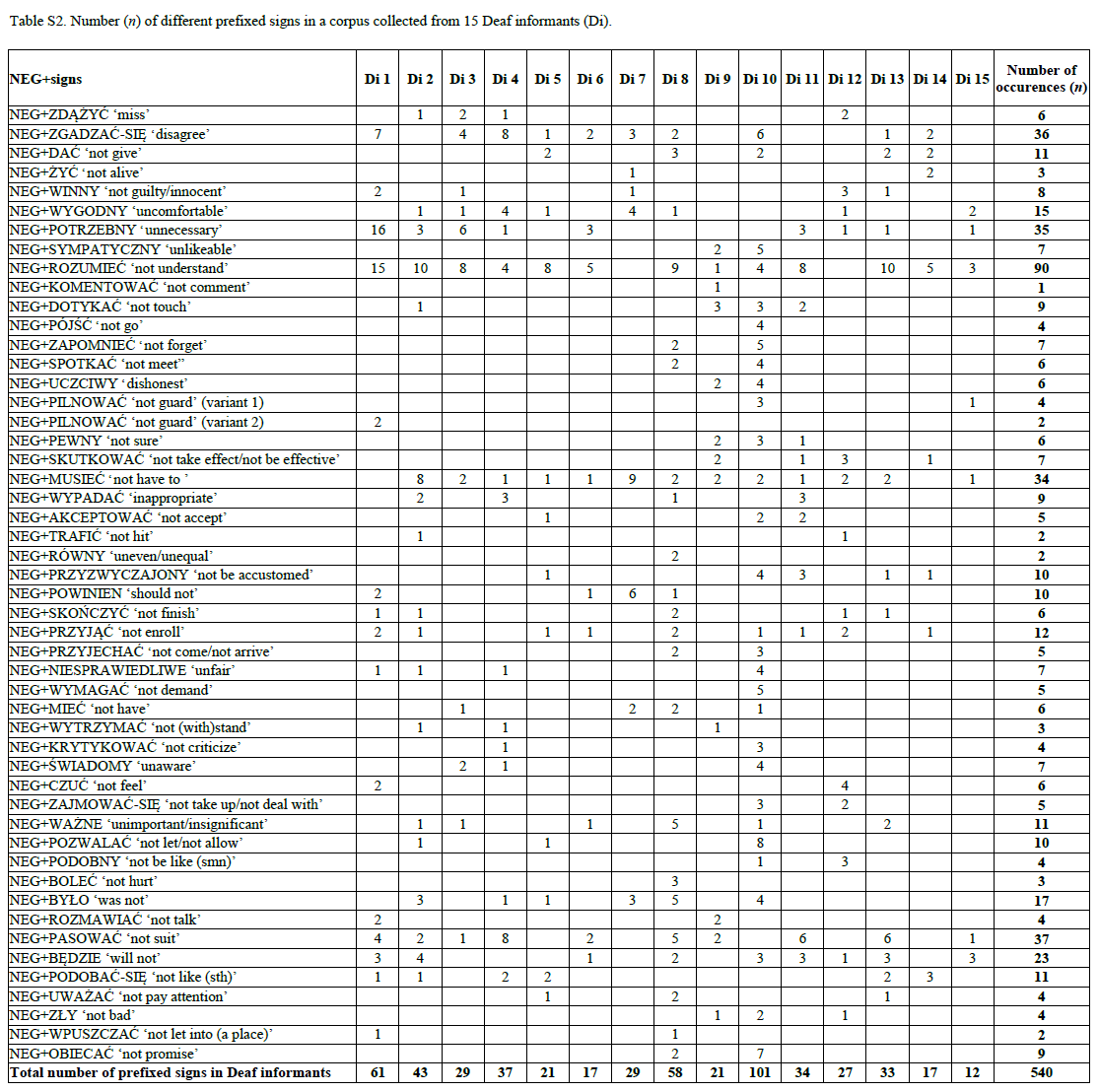

Supplement: S2 Table — (TIF) [file pone.0143574.s006.tif]

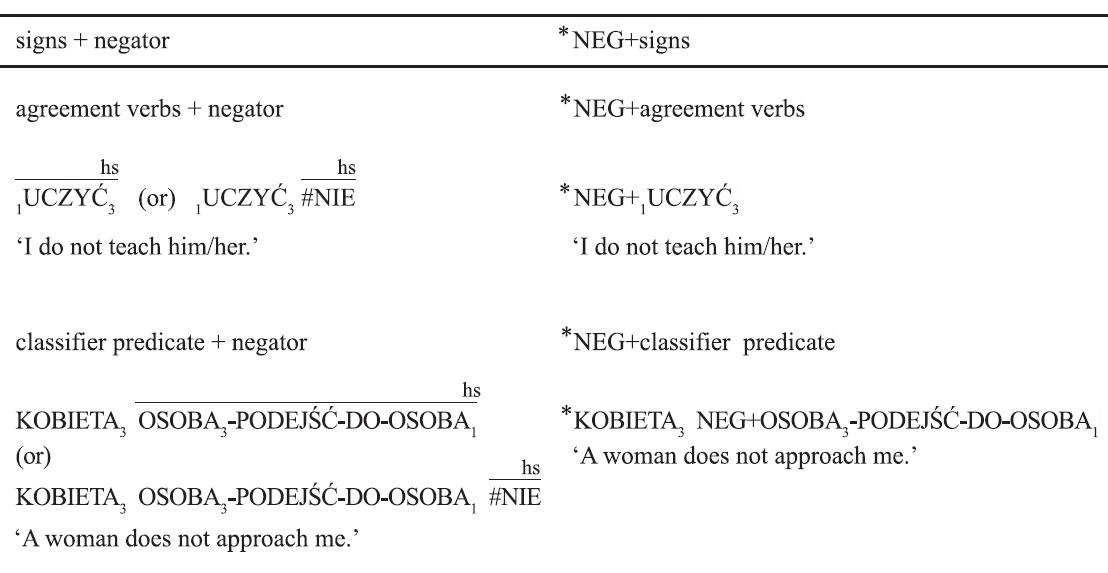

Supplement: S3 Table — hs, non-manual element of negation in the form of a head shake. (TIF) [file pone.0143574.s007.tif]

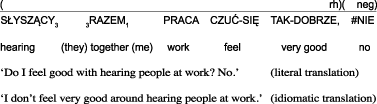

Supplement: S1 Text — In the first part of the sentence shown above in PJM (with Polish words in capital letters standing for PJM signs). There is a rhetorical question (rh) which precedes the final negative element in a stylistically marked way. In this question there are non-manual elements similar to those that occur in yes/no questions, such as raised eyebrows and wide open eyes. However, by the sign TAK-DOBRZE ‘very good’, there is a change in the non-manual elements which sententially apply to the negative expression #NIE. Then the eyebrows are lowered, the nose slightly wrinkles and, most importantly, there is an obligatory head shake (neg). (TIF) [file pone.0143574.s008.tif]
